# New Coleoptera records from New Brunswick, Canada: Buprestidae

**DOI:** 10.3897/zookeys.179.2578

**Published:** 2012-04-04

**Authors:** Reginald P. Webster, Ian DeMerchant

**Affiliations:** 1 Natural Resources Canada, Canadian Forest Service - Atlantic Forestry Centre, 1350 Regent St., P.O. Box 4000, Fredericton, NB, Canada E3B 5P7

**Keywords:** Buprestidae, new records, Canada, New Brunswick

## Abstract

Nine species of Buprestidae; *Agrilus bilineatus* (Weber), *Agrilus crinicornis* Horn, *Agrilus obsoletoguttatus* Gory, *Agrilus putillus putillus* Say, *Brachys ovatus* (Weber), *Buprestis sulcicollis* (LeConte), *Chalcophora liberta* (Germar), *Phaenops aeneola* (Melsheimer), and *Taphrocerus gracilis* (Say) are newly recorded for New Brunswick, Canada. *Agrilus bilineatus*, *A. crinicornis*, *A. obsoletoguttatus*,and *B. ovatus* are also newly reported for the Maritime provinces. Lindgren 12-funnel traps do not appear to be an effective tool for sampling the Bupresidae. Collection, habitat notes, and distribution maps are presented for each species.

## Introduction

Bellamy and Nelson (2002) presented a general overview of the Buprestidae (the metallic wood-boring or jewel beetles) of North America. This species-rich family of beetles is popular with collectors due to their often bright and metallic coloration. Larvae of many of the wood-boring species bore into roots and logs or within bark or cambium layers of trunks or branches of dead or dying trees and shrubs (Bellamy and Nelson 2002). A few species attack living trees and shrubs. Other species are stem and leaf miners of herbaceous and woody plants, including grasses (Bellamy and Nelson 2002). Adults are usually diurnally active, and some species are active strong flyers and often take flight when approached. Adults of some species feed on foliage of their host plants, others feed on pollen or nectar of flowers. Thirty-nine species of Buprestidae were reported from New Brunswick (Bellamy 2008a,b,c; Nelson et al. 2008). Here, we report nine additional species for the province.

## Methods and conventions

The following records are based on specimens collected during a general survey by the first author to document the Coleoptera fauna of New Brunswick and from by-catch samples obtained during a study to develop a general attractant for the detection of Cerambycidae. Additional provincial records were obtained from specimens contained in the collection belonging to Natural Resources Canada, Canadian Forest Service - Atlantic Forestry Centre, Fredericton, New Brunswick.

### Collection methods

Most specimens were collected by sweeping foliage or hand picking from host plants. A few specimens were captured in Lindgren 12-funnel traps during a study to develop a general attractant for the detection of invasive species of Cerambycidae. See Webster et al. (in press) for details of the methods used to deploy Lindgren traps and for sample collection. A description of the habitat was recorded for all specimens collected during this survey. Locality and habitat data are presented exactly as on labels for each record. This information, as well as additional collecting notes, is summarized and discussed in the collection and habitat data section for each species.

### Specimen preparation

Males of some species of Buprestidae (most *Agrilus* species) were dissected to confirm their identity. The genital structures were dehydrated in absolute alcohol and mounted in Canada balsam on celluloid microslides or glued on cards and pinned with the specimens from which they originated.

### Distribution

Distribution maps, created using ArcMap and ArcGIS, are presented for each species in New Brunswick. Every species is cited with current distribution in Canada and Alaska, using abbreviations for the state, provinces, and territories. New records for New Brunswick are indicated in bold under Distribution in Canada and Alaska. The following abbreviations are used in the text:

**Table:** 

**AK**	Alaska	**MB**	Manitoba
**YT**	Yukon Territory	**ON**	Ontario
**NT**	Northwest Territories	**QC**	Quebec
**NU**	Nunavut	**NB**	New Brunswick
**BC**	British Columbia	**PE**	Prince Edward Island
**AB**	Alberta	**NS**	Nova Scotia
**SK**	Saskatchewan	**NF & LB**	Newfoundland and Labrador*

*Newfoundland and Labrador are each treated separately under the current Distribution in Canada and Alaska.

Acronyms of collections examined or where specimens reside referred to in this study are as follows:

**AFC** Atlantic Forestry Centre, Natural Resources Canada, Canadian Forest Service, Fredericton, New Brunswick, Canada

**CNC** Canadian National Collection of Insects, Arachnids and Nematodes, Agriculture and Agri-Food Canada, Ottawa, Ontario, Canada

**NBM** New Brunswick Museum, Saint John, New Brunswick, Canada

**RWC** Reginald Webster Collection, Charters Settlement, New Brunswick, Canada

## Results

### Species accounts

All records below are species newly recorded for New Brunswick, Canada. Species followed by ** are newly recorded from the Maritime provinces of Canada.

The classification of the Buprestidae follows Nelson et al. (2008).

**Table 1.** Species of Buprestidae recorded from New Brunswick, Canada.

**Family Buprestidae Leach**
**Subfamily Chrysochroinae Laporte**
**Tribe Chrysochroini Laporte**
*Chalcophora fortis* LeConte
*Chalcophora liberta* (Germar)*
*Chalcophora virginiensis* (Drury)
*Poecilonota cyanipes* (Say)
**Tribe Dicercini Gistel**
*Dicerca caudata* LeConte
*Dicerca divaricata* (Say)
*Dicerca lugubris* LeConte
*Dicerca tenebrica* (Kirby)
*Dicerca tenebrosa tenebrosa* (Kirby)
*Dicerca tuberculata* (Laporte & Gory)
**Subfamily Buprestinae Leach**
**Tribe Buprestini Leach**
*Buprestis fasciata* Fabricius
*Buprestis maculativentris* Say
*Buprestis striata* Fabricius
*Buprestis sulcicollis* (LeConte)*
**Tribe Anthaxiini Gory & Laporte**
*Anthaxia inornata* (Randall)
*Anthaxia quercata* (Fabricius)
**Tribe Melanophilini Bedel**
*Melanophila acuminata* (DeGeer)
*Phaenops abies* (Champlain & Knull)
*Phaenops aeneola* (Melsheimer)*
*Phaenops drummondi* (Kirby)
*Phaenops fulvoguttatus* (Harris)
**Tribe Chrysobothrini Gory & Laporte**
*Chrysobothris dentipes* (Germar)
*Chrysobothris femorata* (Olivier)
*Chrysobothris harrisi* Hentz
*Chrysobothris neopusilla* Fisher
*Chrysobothris pusilla* Gory & Laporte
*Chrysobothris rotundicollis* Gory & Laporte
*Chrysobothris scabripennis* Gory & Laporte
*Chrysobothris sexsignata* Say
*Chrysobothris trinervia* Kirby
*Chrysobothris verdigripennis* Frost
**Subfamily Agrilinae Laporte**
**Tribe Coraebini Bedel**
*Eupristocerus cogitans* (Weber)
**Tribe Agrilini Laporte**
*Agrilus anxius* Gory
*Agrilus arcuatus* (Say)
*Agrilus bilineatus* (Weber)**
*Agrilus criddlei* Frost
*Agrilus crinicornis* Horn**
*Agrilus cuprescens* (Ménétries)
*Agrilus granulatus liragus* Barter & Brown
*Agrilus obsoletoguttatus* Gory**
*Agrilus pensus* Horn
*Agrilus politus* (Say)
*Agrilus putillus putillus* Say**
*Agrilus ruficollis* (Fabricius)
*Agrilus sayi* Saunders
**Tribe Trachyini Laporte**
*Brachys aerosus* (Melsheimer)
*Brachys ovatus* (Weber)**
*Taphrocerus gracilis* (Say)*

**Notes:** *New to province, **New to Maritime provinces.

### Family Buprestidae Leach, 1815

Nine species of Buprestidae are newly recorded for New Brunswick, Canada. Among these, *Agrilus bilineatus* (Weber), *Agrilus crinicornis* Horn, *Agrilus obsoletoguttatus* Gory, and *Brachys ovatus* (Weber) are also new for the Maritime provinces (New Brunswick, Nova Scotia, Prince Edward Island). Only six specimens of two of the nine species reported here were captured in Lindgren 12-funnel traps during a study to develop a general attractant for the detection of invasive species of Cerambycidae. These traps mimic tree trunks and are often effective for sampling species of Coleoptera that live in microhabitats associated with standing trees (Lindgren 1983). However, the standard black Lindgren funnel traps appear to be much less effective at collecting species of buprestids than species in families such as Cerambycidae, Elateridae, Melandryidae, and many others (see other papers by Webster et al. in this volume). Francese et al. (2011) recently showed mean catch of the invasive emerald ash borer, *Agrilus planipennis* Fairmaire, in Lindgren funnel traps was significantly increased by changing the color from standard black to either purple or green, and by treating the trap surface with Rain-X (ITS Global Brands, Houston, TX), a product normally used to reduce friction and water build-up on windshields. It is possible that use of funnel traps with other colors may enhance the catch of other buprestid species.

### Subfamily Chrysochroinae Laporte, 1835

**Tribe Chrysochroini Laporte, 1835**

#### Chalcophora liberta

(Germar, 1824)

http://species-id.net/wiki/Chalcophora_liberta

Map 1

##### Material examined.

**New Brunswick, York Co.**, Fredericton, 16.VIII.1988, G. J. Crain (1, AFC).

**Map 1.**
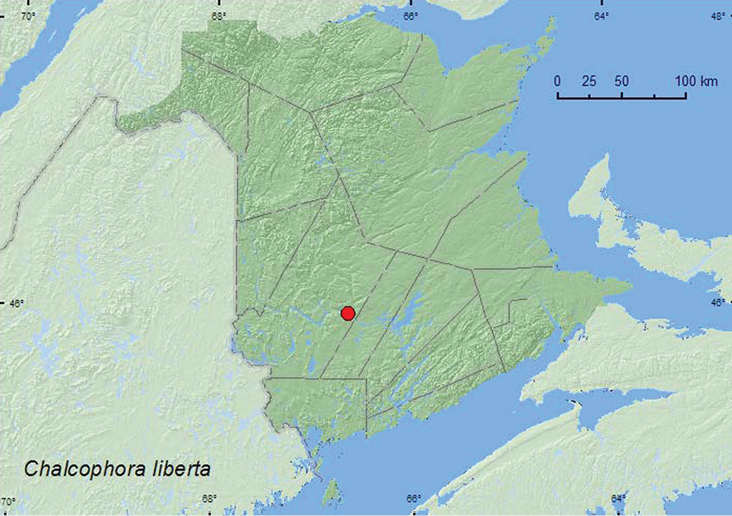
Collection localities in New Brunswick, Canada of *Chalcophora liberta*.

##### Collection and habitat data.

No habitat data were associated with this specimen. Larvae of this species have been reported from red pine (*Pinus resinosa* Ait.) and white pine (*Pinus strobus* L.) (Nelson et al. 2008).

##### Distribution in Canada and Alaska.

MB, ON, QC, **NB**, PE (Bright 1987; Davies 1991; Bellamy 2008a).

### Subfamily Buprestinae Leach, 1815

**Tribe Buprestini Leach, 1815**

#### Buprestis sulcicollis

(LeConte, 1860)

http://species-id.net/wiki/Buprestis_sulcicollis

Map 2

##### Material examined.

**New Brunswick, York Co.**, 3.5 km S jct. Hwy 3 & 4 near Davis Brook, 11.VI.1998, R. P. Webster, on white pine log (1, RWC); 15 km W of Tracy off Rt. 645, 45.6837°N, 66.8809°W, 10.VI.2007, R. P. Webster, clear-cut (old red pine forest), on red pine stump (1, RWC).

**Map 2.**
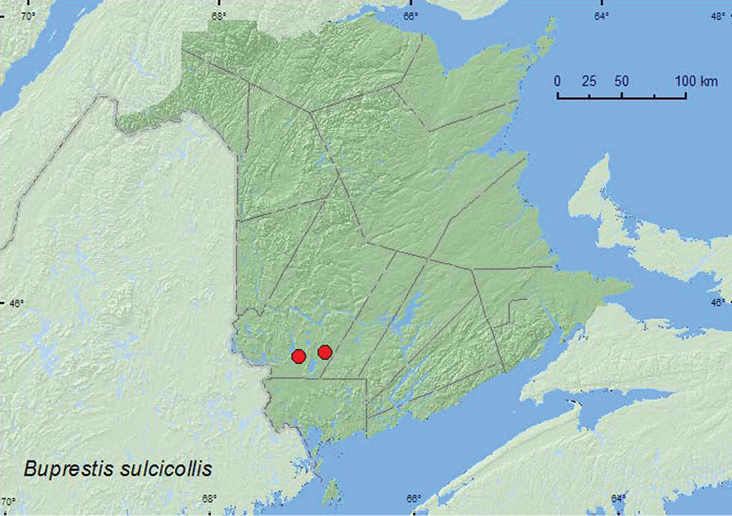
Collection localities in New Brunswick, Canada of *Buprestis sulcicollis*.

##### Collection and habitat data.

Larvae have been reported from pitch pine (*Pinus rigida* Mill.) and white pine (Bright 1987). In New Brunswick, one individual was collected from a white pine log, another from a red pine stump during June.

##### Distribution in Canada and Alaska.

NT, AB, MB, ON, QC, **NB**, NS, NF (Bright 1987; Davies 1991).

### Tribe Melanophilini Bedel, 1821

#### Phaenops aeneola

(Melsheimer, 1845)

http://species-id.net/wiki/Phaenops_aeneola

Map 3

##### Material examined.

**New Brunswick, York Co.**, 15 km W of Tracy off Rt. 645, 45.6848°N, 66.8821°W, 4–11.VIII.2009, R. Webster & M.-A. Giguère, old red pine forest, Lindgren funnel trap (1, AFC); same locality and forest type, emgd. 3–7.V.2010, C. Hughes, reared from small branches of fallen red pine (3, AFC, RWC); same locality and forest type but 27.VII–10.VIII.2010, R. Webster & C. Hughes, Lindgren funnel trap (1, AFC).

**Map 3.**
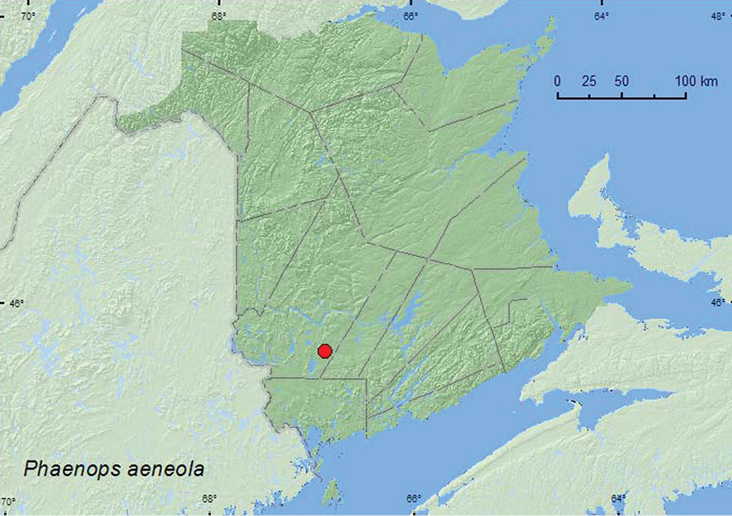
Collection localities in New Brunswick, Canada of *Phaenops aeneola*.<br/>

##### Collection and habitat data.

Larvae of this species have been reported from red pine and Virginia pine (*Pinus virginiana* Mill.) (Nelson et al. 2008). Adults have been reported on jack pine (*Pinus banksiana*), shortleaf pine (*Pinus echinata* P. Mill.), and spruce (*Picea* sp.) (Nelson et al. 2008). In New Brunswick, adults were captured during July and August in Lindgren funnel traps deployed in an old red pine forest. Three adults were reared from small branches of a fallen (during winter 2009) red pine.

##### Distribution in Canada and Alaska.

ON, QC, **NB,** PE (Bright 1987; Davies 1991; Bellamy 2008b).

### Subfamily Agrilinae Laporte, 1835

**Tribe Agrilini Laporte, 1835**

#### Agrilus bilineatus

(Weber, 1801)**

http://species-id.net/wiki/Agrilus_bilineatus

Map 4

##### Material examined.

**New Brunswick, Queens Co.**, Cranberry Lake P.N.A. (Protected Natural Area) 46.1125°N, 65.6075°W, 14.VIII.2009, M.-A. Giguère & R. Webster, red oak forest, on foliage of red oak sapling (1, RWC); same locality data and forest type, 29.VI–7.VII.2011, M. Roy & V. Webster, Lindgren forest trap in forest canopy (4, AFC, NBM, RWC).

**Map 4.**
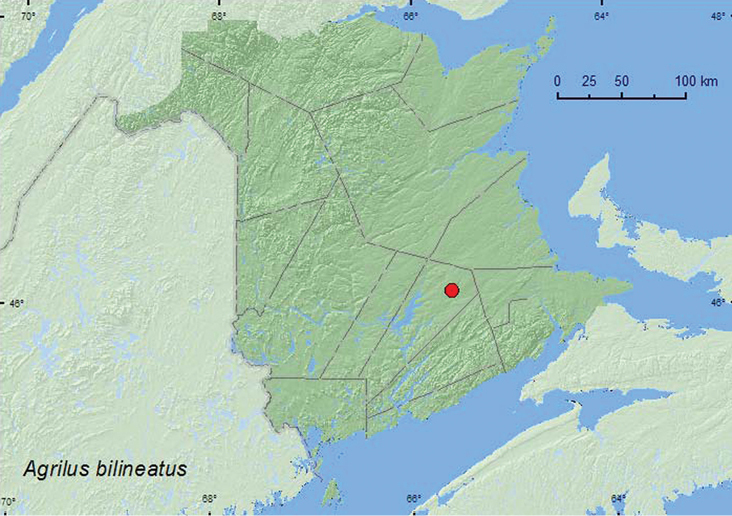
Collection localities in New Brunswick, Canada of *Agrilus bilineatus*.

##### Collection and habitat data.

Larvae of *A. bilineatus* have been reported from a variety of *Quercus* sp., including our native red oak (*Quercus rubra* L.) (Nelson et al. 2008). Adults from New Brunswick were collected during July and August from foliage of red oak and in Lindgren funnel traps deployed in a red oak forest.

##### Distribution in Canada and Alaska.

MB, ON, QC, **NB** (Bright 1987; Davies 1991).

#### Agrilus crinicornis

Horn, 1891**

http://species-id.net/wiki/Agrilus_crinicornis

Map 5

##### Material examined.

**New Brunswick, Queens Co.**, Cranberry Lake P.N.A, 46.1125°N, 65.6075°W, 18.VI.2009, 25.VI.2009, R. Webster & M.-A. Giguère, old red oak forest, on foliage of red oak (3, AFC). **Sunbury Co.**, Burton near Sunpoke Lake, 45.7658°N, 66.5546°W, 20.VI.2007, R. P. Webster, red oak and red maple forest, on foliage of *Quercus rubra* (9, RWC).

**Map 5.**
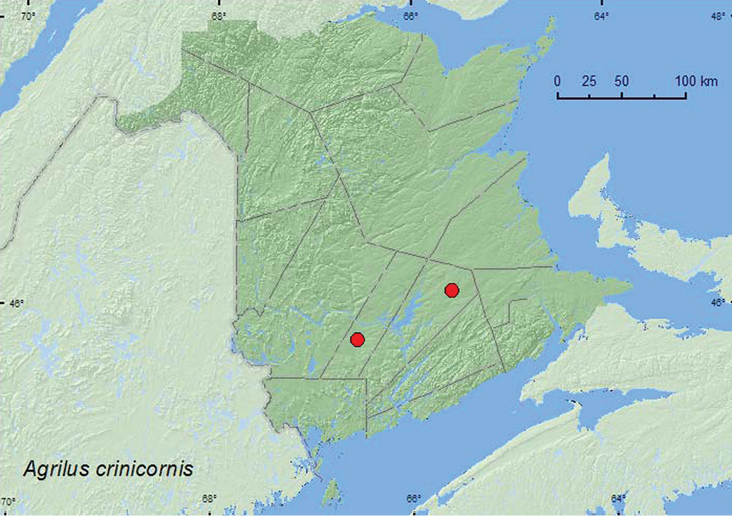
Collection localities in New Brunswick, Canada of *Agrilus crinicornis*.

##### Collection and habitat data.

Larval hosts include *Diospyros virginiana* L., American beech (*Fagus grandifolia* Ehrh.), honey locust (*Gleditsia triacanthos* L.), and white oak (*Quercus alba* L.) (Nelson et al. 2008). *Fagus grandifolia* is the only known host species that occurs in New Brunswick, although other *Quercus* sp. such as *Q. rubra* (red oak) occur in the province. Adults from New Brunswick were collected from foliage of *Q. rubra*, a probable host of *A. crinicornis* in the province.

##### Distribution in Canada and Alaska.

ON, QC, **NB** (Bright 1987; Davies 1991).

#### Agrilus obsoletoguttatus

Gory, 1841

http://species-id.net/wiki/Agrilus_obsoletoguttatus

Map 6

##### Material examined. 

**New Brunswick, Sunbury Co.**, Little Lake Rd., 10.VII.1958 (E. A. Rubridge), on red oak, 58-0795 (2, AFC). (Specimens determined by D.E. Bright, 1981).

**Map 6.**
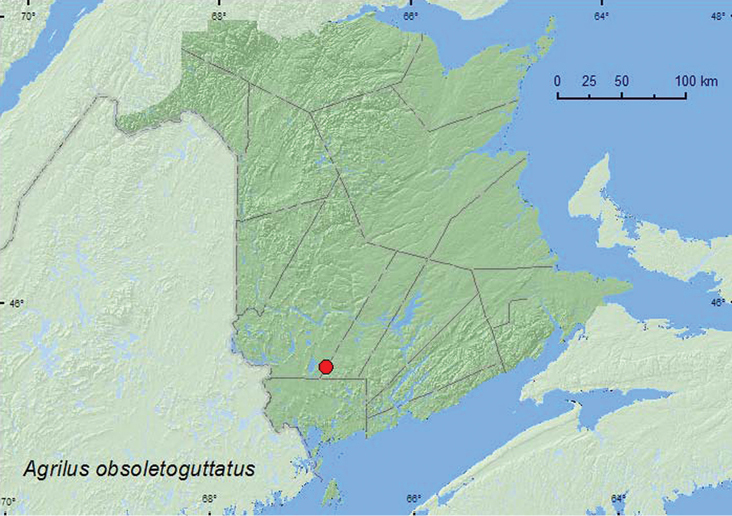
Collection localities in New Brunswick, Canada of *Agrilus obsoletoguttatus*.

##### Collection and habitat data.

Larval hosts of *A. obsoletoguttatus* reported by Nelson et al. (2008) that occur in New Brunswick include red oak, ironwood (*Ostrya virginiana* (Mill.) K. Koch)), and *Fagus* sp. The specimens from New Brunswick were collected from foliage of red oak during July.

##### Distribution in Canada and Alaska.

ON, QC, **NB** (Bright 1987; Davies 1991).

#### Agrilus putillus putillus

Say, 1833

http://species-id.net/wiki/Agrilus_putillus_putillus

Map 7

##### Material examined.

**New Brunswick, Carleton Co.**, Jackson Falls, Bell Forest, 46.2200°N, 67.7231°W, 8.VII.2008, R. P. Webster, Rich Appalachian hardwood forest, m.v. light (1, RWC).

**Map 7.**
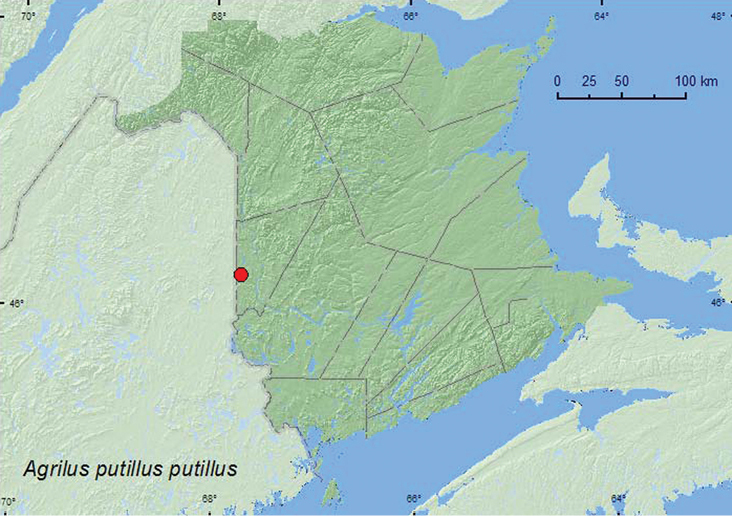
Collection localities in New Brunswick, Canada of *Agrilus putillus putillus*.

##### Collection and habitat data.

Larval hosts include sugar maple (*Acer saccharum* Marsh.), Norway maple (*A. platanoides* L.), and honey locust (Nelson et al. 2008). The adult from New Brunswick was collected during July at a mercury vapor light in a forest with sugar maple, American beech, and white ash (*Fraxinus americana* L.), and other hardwood species.

##### Distribution in Canada and Alaska.

ON, QC, **NB**, PE(Bright 1987; Davies 1991; Bellamy 2008c).

### Tribe Trachyini Laporte, 1835

#### Brachys ovatus

(Weber, 1801)**

http://species-id.net/wiki/Brachys_ovatus

Map 8

##### Material examined. 

**New Brunswick, Sunbury Co.**, Burton near Sunpoke Lake, 45.7659°N, 66.5563°W, 28.VII.2007, R. P. Webster, margin of red oak stand near lakeshore, on foliage of *Quercus rubra* (1, RWC).

**Map 8.**
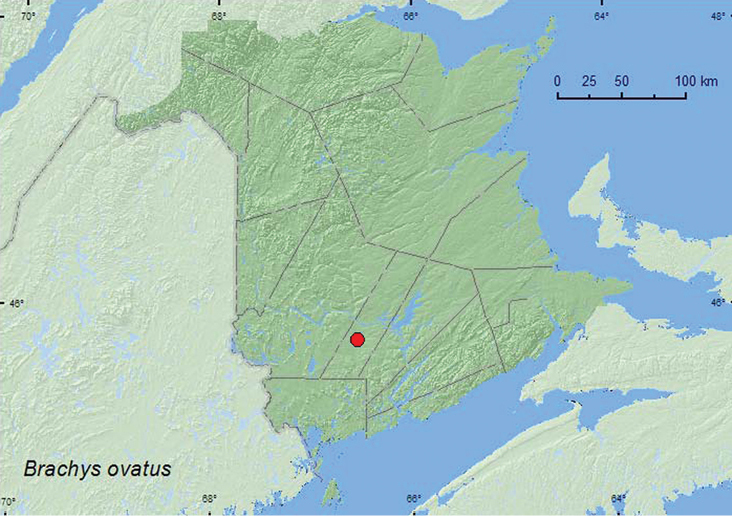
Collection localities in New Brunswick, Canada of *Brachys ovatus*.

##### Collection and habitat data.

Hosts include avariety of *Quercus* sp., including red oak (Nelson et al. 2008). One adult from New Brunswick was collected in late July from foliage of red oak.

##### Distribution in Canada and Alaska.

ON, QC, **NB** (Bright 1987; Davies 1991).

#### Taphrocerus gracilis

(Say, 1825)

http://species-id.net/wiki/Taphrocerus_gracilis

Map 9

##### Material examined. 

**New Brunswick, York Co.**, Charters Settlement, 45.8428°N, 66.7279°W, 20.IV.2005, R. P. Webster, mixed forest, small sedge marsh, in moist grass litter & sphagnum (1, RWC); same locality and collector but 45.8430°N, 66.6275°W, 17.VI.2007, regenerating mixed forest, sweeping foliage of *Carex* species in small marshy area (1, RWC); 17 km W of Tracy off Rt. 645, 45.6816°N, 66.9060°W, 2.VII.2008, R. P. Webster, red pine forest, marshy area in roadside ditch, sweeping (1, RWC).

**Map 9.**
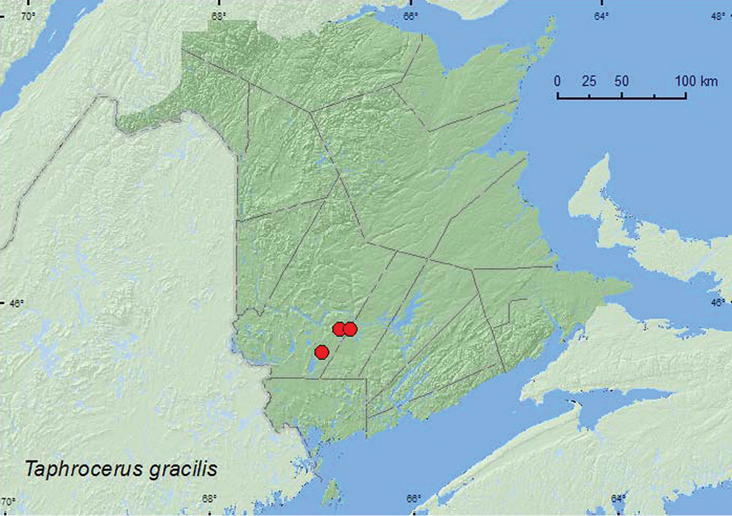
Collection localities in New Brunswick, Canada of *Taphrocerus gracilis*.

##### Collection and habitat data.

Larval hosts include beak-rush (*Rhynchospora corniculata* (Lam.)) and bulrush (*Schoenoplectus fluviatilis* (Torr.)) (Nelson et al. 2008). Although the above host species do not occur in New Brunswick, related species in these genera occur in the province (Hinds 2000). Adults have been reported from *Carex hyalinolepus* Steud., buttonbush (*Cephalanthus occidentalis* L.), and dock (*Rumex verticillatus* L.). Adults from New Brunswick were collected from *Carex* sp., swept from foliage in a marshy area in a roadside ditch, and sifted from moist grass litter and sphagnum in a small *Carex* marsh. Adults were captured during April, June, and July.

##### Distribution in Canada and Alaska.

AB, SK, MB, ON, QC, **NB**, NS (Bright 1987; Davies 1991; Bellamy 2008c).

## Supplementary Material

XML Treatment for Chalcophora liberta

XML Treatment for Buprestis sulcicollis

XML Treatment for Phaenops aeneola

XML Treatment for Agrilus bilineatus

XML Treatment for Agrilus crinicornis

XML Treatment for Agrilus obsoletoguttatus

XML Treatment for Agrilus putillus putillus

XML Treatment for Brachys ovatus

XML Treatment for Taphrocerus gracilis
